# Sex-Specific Selection and Sex-Biased Gene Expression in Humans and Flies

**DOI:** 10.1371/journal.pgen.1006170

**Published:** 2016-09-22

**Authors:** Changde Cheng, Mark Kirkpatrick

**Affiliations:** Department of Integrative Biology, University of Texas, Austin, Texas, United States of America; University of California, Berkeley, UNITED STATES

## Abstract

Sexual dimorphism results from sex-biased gene expression, which evolves when selection acts differently on males and females. While there is an intimate connection between sex-biased gene expression and sex-specific selection, few empirical studies have studied this relationship directly. Here we compare the two on a genome-wide scale in humans and flies. We find a distinctive “Twin Peaks” pattern in humans that relates the strength of sex-specific selection, quantified by genetic divergence between male and female adults at autosomal loci, to the degree of sex-biased expression. Genes with intermediate degrees of sex-biased expression show evidence of ongoing sex-specific selection, while genes with either little or completely sex-biased expression do not. This pattern apparently results from differential viability selection in males and females acting in the current generation. The Twin Peaks pattern is also found in *Drosophila* using a different measure of sex-specific selection acting on fertility. We develop a simple model that successfully recapitulates the Twin Peaks. Our results suggest that many genes with intermediate sex-biased expression experience ongoing sex-specific selection in humans and flies.

## Author Summary

Sexual dimorphism, which is evident in virtually all phenotypic traits, results from sex-biased gene expression. The evolution of sex-biased expression, in turn, results from selection that acts differently on males and females. We use genomic data from humans and flies to quantify the relation between ongoing selection pressures and gene expression. A distinctive pattern in both species reveals that many genes are experiencing selection that is currently acting differentially on males and females. These results suggest that the “war between the sexes” is more than a metaphor: it contributes to observable patterns of mortality in humans.

## Introduction

Females and males differ for virtually all phenotypic traits [1]. Sexual dimorphism results from sex-biased gene expression, which evolves in response to selection that acts differently on males and females [2–4]. Thus sex-biased gene expression is intimately linked to sex-specific selection [5,6]. What is less clear, however, is the extent to which sex-specific selection is ongoing, and how ongoing selection relates to the strength of sex-biased expression.

To date, the most direct link between sex-specific selection and sex-biased expression comes from a laboratory study of *Drosophila melanogaster*. By comparing gene expression and reproductive fitness in a quantitative genetics design, Innocenti and Morrow [7] identified genes experiencing sexually-antagonistic selection, which is the extreme case of sex-specific selection in which an allele that increases fitness in one sex decreases it in the other. They concluded that 8.5% of loci that show sex-biased expression are experiencing ongoing sexually-antagonistic selection. While this work is a milestone, it leaves key questions unanswered. These include: What is the relation between the strength of ongoing sex-specific selection and sex-biased expression, and how common and how strong is sex-specific selection in natural populations? Answers to these questions are important to our understanding of how sexual dimorphism evolves. Further, the answers will inform us about general issues including constraints to adaptation [8–10] and how genomes evolve [11–13].

We tackle this problem here with a new method that directly quantifies contemporary sex-specific selection on a genomic scale. We reason that Mendelian inheritance ensures that allele frequencies at autosomal loci are equal in males and females at conception, and that sex-specific viability selection will generate genetic divergence between the sexes within a generation. Divergence will occur when selection is sexually-antagonistic, meaning that different alleles are favored in males and females. Divergence also results when selection of different strengths favors the same allele in males and females, a situation sometimes called “sexually-concordant selection” [14]. We use the term “sex-specific selection” here to include both cases.

We find that genes with intermediate degrees of sex-biased expression experience the strongest sex-specific selection in humans. We assess the generality of this pattern by reanalyzing the data on flies from Innocenti and Morrow [7]. We again find the The Twin Peaks pattern, which in this case results from sexually-antagonistic (not sexually-concordant) selection, and from selection on fertility (rather than viability). A simple population-genetic model successfully recapitulates the key pattern. Our results suggest that ongoing sex-specific selection is a common feature of the genome in humans and flies.

## Results

### Sex-specific selection and sex-biased expression in humans

We quantify sex differences in viability selection using *F*_ST_ between adult males and females [15]. We used allele frequencies at over 6 million autosomal single-nucleotide polymorphisms (SNPs) that appear in the 1000 Genomes Project [16], a database that includes more than 2000 individuals from 26 populations worldwide. For each of 17,839 protein-coding loci, we calculated the average *F*_ST_ for all SNPs within transcribed regions within each population. We expect this to give a conservative picture of sex differences in selection since most SNPs are not themselves targets of selection. Rather, they change frequency through hitchhiking, and the *F*_ST_ that results at these SNPs is expected to be less than that at a selected SNP in the same gene. Patterns that are qualitatively the same as what we report below also emerge if we use only the single SNP from each locus with the largest value of *F*_ST_. No SNP showed significantly different allele frequencies in males and females at a false discovery rate of 5%. That is not surprising, however. A power analysis presented in the S1 File shows it is highly unlikely to observe significant divergence at any individual SNP, even with very strong sexually-antagonistic selection, simply because the correction for multiple comparisons is so severe.

We quantify sex-biased expression as the normalized difference between expression in males and females: Δ = (*m*–*f*) / (*m* + *f*), where *m* and *f* are the transcript abundances in males and females. (This measure is highly correlated with the familiar log_2_ of the expression ratio.) A value of Δ = –1 means a gene is expressed only in females, Δ = 0 means expression is unbiased, and Δ = 1 means it is expressed only in males. We use RNAseq data from the Genotype-Tissue Expression Project [17]. Sex-biased expression in adults mainly results from differences between reproductive tissues [18,19], so we used expression levels in testis and ovaries. The data are from 14 males and 6 females [20].

**Fig 1:**
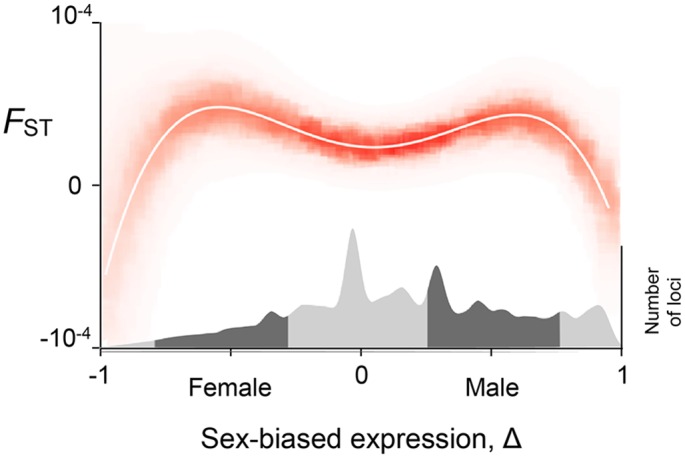
The strength of sex-specific selection is strongest on human autosomal genes with intermediate sex-biased expression. The white curve is the best-fit 4^th^ degree polynomial and the intensity of red indicates the likelihood that the regression passes through a given value. The average *F*_ST_ for the SNPs in a gene is small at Δ = 0 (unbiased expression), increases to a peak as sex-bias grows, then decreases as Δ approaches –1 and 1. The numbers of genes with a given bias are visualized in the density plot in the lower part of the figure; dark gray denotes intermediate sex-biased expression.

We find that genetic divergence between males and females is related to the degree of sex-biased expression by a distinctive pattern that we call “Twin Peaks” (Fig 1). Average *F*_ST_ reaches a minimum when expression is unbiased (Δ = 0). Genetic divergence between the sexes is greatest for genes whose expression is moderately female-biased $\left(\Delta \approx –\frac{1}{2}\right)$ or moderately male-biased $\left(\Delta \approx \frac{1}{2}\right)$. After reaching these peaks, genetic divergence then declines again as expression tends towards complete female-bias (Δ = –1) and complete male-bias (Δ = 1).

The Twin Peaks pattern is statistically well-supported. The best fit regression of *F*_ST_ on Δ is a fourth-degree polynomial with two peaks (S1 Table). We permutated the data 10^5^ times and found the pattern occurs 1.6% of the time (that is, the pattern is significant at *p* = 0.016, details in Materials and Methods). The Twin Peaks pattern is also recovered by fitting a cubic spline using generalized additive models. Twin Peaks are seen within each of the 26 populations when they are analyzed separately. Genetic divergence between the sexes is quite repeatable across populations: taking the SNP with highest *F*_ST_ at each locus, the intra-class correlation coefficient is 0.34. Finally, the Twin Peaks also emerge when we measure genetic divergence between the sexes using the statistic *D*_a_ instead of *F*_ST_ [21,22] (S2 Table, S3 Fig).

It is plausible that gene expression in adult gonads (on which our analyses are based) is not itself the target of the viability selection that causes the Twin Peaks pattern. Instead, expression in adult gonads could be correlated with expression in other tissues and other life stages that are the actual targets. Unfortunately, we are not able to repeat our analyses with most somatic tissues because the Gene Expression Atlas only provides data on expression averaged across both sexes. We were however able to analyze expression in five sex-specific somatic tissues: ectocervis, fallopian tubes, vagina, and uterus in females, and prostate in males. The Twin Peaks pattern appears again when we use the average expression of the four female-specific somatic tissues and expression in testes, but not when we use prostate instead of testes. Expression in prostate may not provide a good proxy for gene expression in males, however, as its expression profile is very similar to that of vagina and other female-specific tissues [20].

Sex-specific selection could be acting on regulatory elements as well as coding regions. We therefore repeated the analyses including all noncoding transcripts whose expression is profiled in the Genotype-Tissue Expression Project (34,060 in total) and the 1kb sequence upstream of all coding regions used in the previous analysis. Once again, a significant Twin Peaks pattern appears.

We considered the possibility that the pattern is driven by heterozygosity. Imagine that the strength sex-specific selection does not vary systematically with sex-biased expression, but that heterozygosity follows the Twin Peaks pattern. Then genetic divergence between males and females, measured as *F*_ST_, will also show that pattern. (This prediction follows because changes in allele frequencies caused by selection are proportional to heterozygosity.) We therefore regressed the quantity (*F*_ST_/*pq*) onto Δ, where *p* and *q* are the frequency of the two alleles at a given SNP. If the Twin Peaks results from variation of heterozygosity with sex-biased expression, the regression is expected to be flat. This alternative hypothesis is falsified: Twin Peaks are again seen using the polynomial and spline regressions described above (S3 Table). We also fit a regression model that included both heterozygosity and Δ as factors. The Twin Peaks pattern remains significant (*p* < 0.01) even after the effect of heterozygosity on *F*_ST_ is controlled for.

While no individual SNP showed significant *F*_ST_ between the sexes, the identities of several highly diverged genes do hold hints of possible connections to sex-specific selection. The gene *RNF212* segregates for alleles that have opposing effects on male and female recombination rates [23,24]. The mean *F*_ST_ for the SNPs in this gene is in the top 0.2% of all the loci we analyzed. Other genes in the top 5% of *F*_ST_ values are two loci (*LYPLAL1* and *ADAMTS9*) associated with the waist-hip ratio [25]. This is a sexually-dimorphic phenotype that has long been associated with sexual attractiveness [26]. A recent review identified 33 loci that have been linked by multiple genome-wide association studies to sex-specific risk of disease [27]. The most diverged SNPs at these genes have *F*_ST_ values that are significantly higher than average (*p* < 4 x 10^−5^ by a permutation test). A final observation is that a set of genes that show evidence of balancing selection [28] also have *F*_ST_ values that are significantly higher than the genome-wide average (*p* < 1 x 10^−6^ by a permutation test). This correlation could result if sexually-antagonistic selection itself is maintaining polymorphism at these loci [29], or if sex-specific selection acts on polymorphisms that are maintained by other mechanisms (e.g. overdominance).

These results lead us to conclude that the Twin Peaks pattern in humans results from sex-specific viability selection whose strength varies with the degree of sex-biased gene expression.

### Twin Peaks also occur in flies

To test the generality of the pattern we discovered in humans, we reanalyzed the fly data from Innocenti and Morrow [7]. Here our measure of the strength of sex-specific selection acting on a locus is binary: it takes a value of 1 if they identified it as a target of sexually-antagonistic selection, and is 0 otherwise. Their data are based on fertility selection, rather than viability selection. We again quantified sex-biased gene expression using Δ based on their transcriptome data. We then asked if fraction of genes under sexually-antagonistic selection varies systematically with Δ.

**Fig 2:**
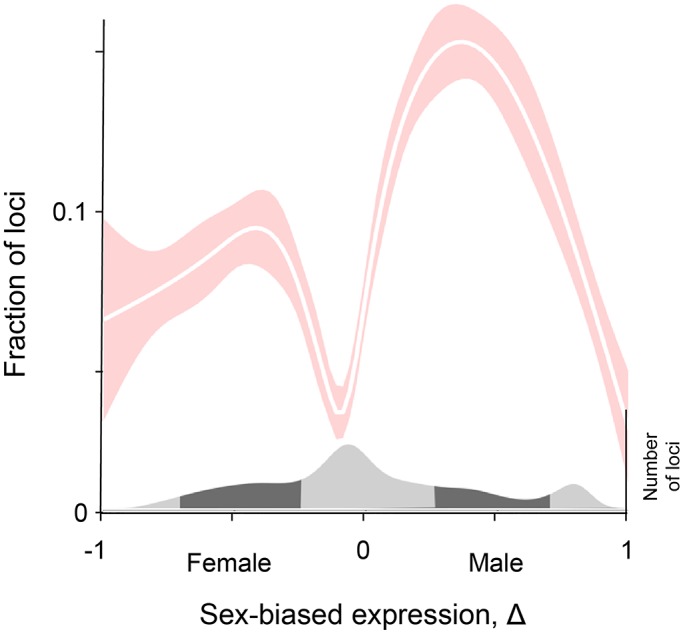
The Twin Peaks pattern in flies. The relation between the fraction of genes under sexually-antagonistic selection and Δ is shown by the best-fit cubic spline (white line), and the 95% confidence interval is shown by the shaded areas. The distribution of gene numbers is shown at the bottom of the figure.

The Twin Peaks pattern appears once again (Fig 2). The greatest fraction of loci under sexually-antagonistic selection have intermediate sex-biased expression. Only a small fraction of genes experience sexually-antagonistic selection when expression is not sex-biased (Δ = 0) or when it is completely sex-biased (Δ = –1 or 1). The pattern is highly statistically significant (*p* < 10^−5^) by bootstrapping using regressions based both on polynomials and splines (see Materials and Methods).

The pattern in flies is consistent with that in humans. A difference between the two data sets is that the pattern in humans results from genetic effects on viability, while the pattern in flies reflects effects on fertility and fecundity. Together, these results suggest that a tendency for stronger sex differences in selection to act on genes with intermediate sex-biased expression may be general to diverse forms of selection.

### A simple model explains Twin Peaks

**Fig 3:**
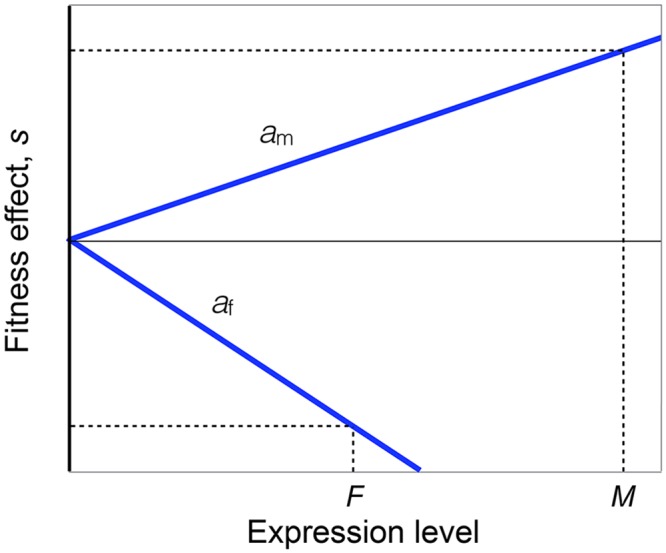
Schematic of the genetic model. The X-axis shows the expression of a locus, measured as the log of the number of transcripts, in males (*M*) and females (*F*). The Y-axis shows the additive fitness effect of an allele in males (*s*_m_) and females (*s*_f_). No selection (*s*_m_, *s*_f_ = 0) occurs when there is no expression (*M*, *F* = 0). Fitness effects increase with expression at a rate *a*_m_ in males and *a*_f_ in females. At an evolutionary equilibrium, *s*_m_ = –*s*_f_. Our measure Δ of sex-bias is approximately equal to twice the difference between *M* and *F*, normalized by the total of expression in both sexes, *M* + *F*, when Δ is small. Further details are given in the Materials and Methods section.

To understand the Twin Peaks pattern, we built a simple model that relates gene expression to fitness. Three key assumptions are invoked: (1) Allele frequencies are at equilibrium under sexually-antagonistic viability selection, which is the special case of sex-specific selection in which selection favors one allele in one sex and the other allele in the other sex; (2) A gene that is not expressed experiences no selection; and (3) At low expression levels, the effects of alleles on viability increase approximately linearly with the amount of expression. These assumptions are illustrated graphically in Fig 3. The model allows selection to be frequency-dependent, and for arbitrary dominance. Further details and the analysis of the model are given in the Materials and Methods section.

This model leads to two predictions regarding how genetic divergence between the sexes varies with the degree sex-biased expression. First, we find that when Δ is near to 0,

$$F_{\text{ST}}\approx 4\;p\;q{\left(\frac{a_{m}a_{f}}{a_{m}+a_{f}}\right)}^{2}\Delta^{2},$$ (1)

where *a*_m_ and *a*_f_ measure how the strength of selection varies with the gene’s expression in males and females (see Fig 3). We see that allele frequency divergence between the sexes depends on three kinds of quantities: heterozygosity (proportional to 4*pq*), the potential for sexually-antagonistic selection (represented by the term in parentheses), and the strength of sex-biased expression (represented by Δ).

**Fig 4:**
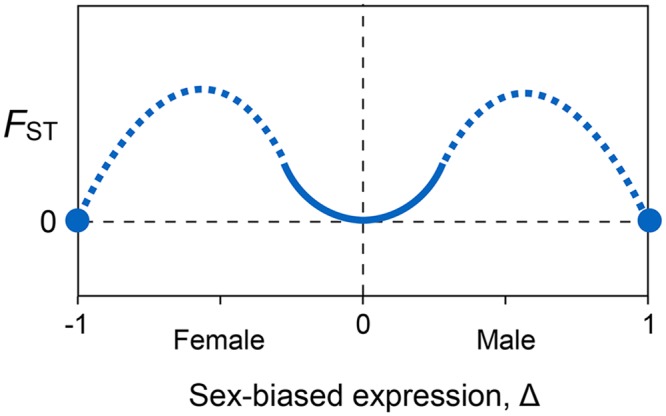
Qualitative conclusions from the genetic model. Genetic divergence between the sexes, measured as *F*_ST_, is minimized when gene expression is unbiased (Δ = 0), then increases quadratically for small degrees of female-biased and male-biased expression (solid curve). *F*_ST_ is also 0 when expression is completely female-biased (Δ = –1) or male-biased (Δ = 1) (solid circles). Values of *F*_ST_ expected for intermediate sex-dependent expression are interpolated by eye (dashed curves).

All else equal, this result predicts that *F*_ST_ will be 0 when expression is unbiased, and it will increase quadratically as expression becomes slightly female- or male-biased. Second, the model predicts that *F*_ST_ will also be zero when expression is completely female-biased and completely male-biased. The model does not apply to genes whose expression is strongly but not completely sex-biased. We can, however, make qualitative predictions for these cases by interpolating between the predictions from the first two cases. The pattern that results is Twin Peaks (Fig 4).

To check the qualitative fit of this model to data, we focused on genes in the human dataset whose expression is weakly sex-biased. We took all genes expression lies between the two peaks at Δ = –0.52 and Δ = 0.72, which includes 80% of the genes in the dataset. The best-fit polynomial regression is a quadratic with a significantly positive coefficient (*p* << 0.001), as predicted by our model (S4 Table). In principle, we might use the curvature of this regression to estimate the quantity that appears within parentheses in Eq (1). That would be of interest because it is a measure of the potential for sexually-antagonistic selection. We do not take that step here, however, because as we now explain these data may not be adequate to give accurate estimates of selection.

### The strength and prevalence of sex-specific selection in humans

Under the assumptions of our model, the value of *F*_ST_ between males and females is proportional to the strength of sexually-antagonistic viability selection (see Materials and Methods). This suggests that one might estimate the strength of sexually-antagonistic selection from these data. Using the average *F*_ST_ for a gene biases the estimate downward because the large majority of SNPs are not expected to be direct targets of selection. Using the SNP in a gene with the largest *F*_ST_, on the other hand, biases estimates upward because this extreme value is expected to result partly from sampling variance.

With these caveats in mind, we attempted to make a rough estimate for the strength of sexually-antagonistic selection acting on genes with intermediate sex-biased expression (see Materials and Methods). Focusing on the YRI population (Yoruba in Ibadan, Nigeria) because it is the most genetically variable, we sampled loci whose values of Δ fall close to maxima of the two Twin Peaks and whose values of *F*_ST_ are close to the average for genes with that degree of sex-biased expression. Using the average *F*_ST_ for all the SNPs in each gene, we estimate that the additive fitness effects of alleles at these loci are *s* = 0.02 on average. If correct, this result would suggest that sexually-antagonistic selection at these loci is strong. The estimate is based on small numbers of individuals, however, and so it is imprecise. The estimate is also likely to be biased upwards, as we will see shortly.

What fraction of genes experiences sex-specific selection in humans? While it is difficult to quantify that number with precision, we can get a crude idea by summing the number of loci that lie beneath the Twin Peaks, for example those whose sex-biased expression lies between 0.25 < | Δ | < 0.75. In both humans and flies, those are about 40% of coding genes. These results suggest that sex-specific selection could be common in both species.

### The selection load

Sex differences in autosomal allele frequencies between mature females and males result from differential survival after conception. Unlike other kinds of genetic load [30], this engenders a true mortality load on the population. We can calculate this load under the assumption of our model that the loci are at equilibrium under sexually-antagonistic selection in which an allele that increases viability in one sex decreases it in the other (see Materials and Methods). The additional mortality incurred by a population due to sexually-antagonistic selection on a single locus with additive effects is simply *L*_SA_ = *s*, where *s* is the fitness effect of an allele (positive in one sex, negative in the other). This result suggests that not many loci can be under strong sexually-antagonistic selection or the population would not be viable. For example, sexually-antagonistic acting on 100 loci with independent fitness effects of *s* = 0.01 reduces the average viability in the population by 63%.

This consideration argues that the estimates for the strength and prevalence of selection offered in the previous section may be substantial overestimates. Factors that may contribute are discussed below.

## Discussion

This study focuses on the relation between the strength of ongoing sex-specific selection to the degree of sex-biased expression. In humans, we used allele frequencies at 6 million coding SNPs sampled from 26 populations. We quantify the strength of sex differences in selection acting within a generation using *F*_ST_ between males and females adults. In flies, we quantified sex-specific selection as the fraction of loci that had been previously identified as targets of sexually-antagonistic selection [7]. In both species, we see a distinctive Twin Peaks pattern in which sex-specific selection is strongest for genes with intermediate sex-biased expression, and is weak when sex-bias expression is either complete or absent. These results suggest that sex-specific selection is ongoing at many genes. We develop a simple genetic model that recapitulates the Twin Peaks pattern.

In a recent study, Lucotte *et al*. [31] used the *F*_ST_-based strategy that we employed to scan the HapMap dataset [32] for SNPs with divergent allele frequencies in males and females. Under their most stringent statistical criteria, they found two X-linked loci that are significantly diverged (at a FDR of 5%). (Those loci do not appear in our analyses because we excluded the sex chromosomes.) Further, they reported that extreme *F*_ST_ values are enriched on the X chromosome relative to the autosomes, consistent with what is expected from sexually-antagonistic selection. Taken together with our results from the 1000 Genomes dataset, the consistent picture is that there is evidence of ongoing sex-specific selection in humans.

Other processes besides sex-specific selection might contribute to the sex differences in allele frequencies we observed in humans. Population structure can generate *F*_ST_ between the sexes. If populations have different allele frequencies, then differential migration of one sex will generate *F*_ST_ between the sexes within a population. *F*_ST_ between the sexes will also appear in the data if viability selection acts equally on males and females but the sexes are sampled at different ages.

It seems unlikely, however, that these alternative hypotheses explain the Twin Peaks pattern. While both population structure and age differences can inflate *F*_ST_ between the sexes in the human dataset, it is difficult to see how either of those factors would generate a pattern that varies systematically with sex-biased expression. Further, neither population structure nor age differences are factors in the fly dataset. Sex-specific selection therefore appears to be strongly supported.

As we noted in the Results, it is plausible that the Twin Peaks pattern does not result from viability selection on gene expression in adult gonads (which is what our analysis is based on), but rather from selection on correlated traits such as expression in other tissues and/or at other ages. If so, we expect that our results are conservative: the pattern we observed is weaker than what would be seen if divergence in male and female allele frequency was regressed against the true targets of selection.

There is good reason, however, to think that our estimates for the strength of selection and the selection load in humans are biased upwards. The calculations assume loci are at equilibrium under sexually-antagonistic selection in which selection in one sex is exactly counterbalanced by selection in the other. If instead sex-specific selection acts on polymorphisms that are maintained by other forces (e.g. migration or mutation) or that are transient, then the load could be substantially lower. Unfortunately, it does not seem possible to estimate the strength of selection from our data under these more general conditions. Further, as we discussed earlier, geographical and age structure in the data may contribute to the genetic differences between the sexes.

The fly data also include biases. One results from sex differences in the allometry of body parts. Innocenti and Morrow [7] measured gene expression in the whole bodies of flies. Differences in transcript abundances between tissues that have different relative sizes in males and females will result in sex differences in relative expression, and so bias our Δ statistic. It is difficult, however, to see how this bias would vary systematically with the fraction of genes under sexually-antagonistic selection and so produce the Twin Peaks pattern.

Our model suggests that the Twin Peaks pattern is expected to emerge under certain conditions. Two in particular deserve consideration. The first assumption is that selection is sexually-antagonistic, with different alleles favored in males than in females. Our *F*_ST_-based analysis of the human data is not able to discriminate between sexually-antagonistic selection and sexually-concordant selection (when the same allele is favored with different strengths of selection in males and females). We note, however, that the Twin Peaks pattern in flies does result from sexually-antagonistic (and not sexually-concordant) selection. Second, the model assumes that allele frequencies are at equilibrium. Unfortunately, we are unable to generalize either of these two assumptions. But while the quantitative results from the model will not hold if those assumptions are violated, it seems likely that the Twin Peaks pattern will emerge under a broader range of conditions than our model assumes.

Our results and those of Innocenti and Morrow [7] suggest that a substantial number of genes are targets of ongoing sex-specific selection. Why hasn’t evolution adjusted expression levels and resolved this tension? Two general ideas have been offered. One is that pleiotropy is extensive and so there is little genetic variation available to decouple expression levels in males and females [2,33–36]. A second suggestion is that selection pressures may fluctuate over time and space, and evolutionary lags prevent the degree of sex-biased expression from being optimized in the current environment [37–39]. This hypothesis is consistent with the high turnover rate seen of genes with sex-biased expression [19,40–42].

The holy grail of evolutionary genomics has been to identify individual genes that underlie adaptation. Our approach shows that even when that goal is not possible, we can nevertheless make important inferences about selection and adaptation by studying genome-wide patterns. The strategy of combining data across genes with a simple model has offered a new perspective on the evolution of sex-biased gene expression. Similar strategies may also be fruitful in studies of other problems.

## Materials and Methods

### A simple model relating *F*_ST_ between the sexes to sex-biased gene expression

To understand the relation between sex-biased expression and sex-specific selection, we developed a very simple model. We reasoned that sex-specific selection at a locus must vanish when it is not expressed in one of the sexes. We approximate the strength of viability selection acting on two alleles in males and females as functions that are linear with the relative expression levels and that have intercepts at the origin (Fig 3). We further assume that allele frequencies are at equilibrium under sexually-antagonistic selection, such that selection favoring an allele in one sex is balance by selection against that allele in the other sex. We will now use this visualization to make a quantitative connection between sex-biased expression and genetic divergence between the sexes.

Allele frequencies at autosomal loci are equal in males and females among zygotes. Sexually-antagonistic selection acting on viability will cause divergence in allele frequencies between adult males and females. Consider a locus segregating for alleles *A* and *a*. To measure the impact of sexually-antagonistic selection, we use the familiar *F*_ST_ statistic:

$$F_{ST}=\frac{{\left(p_{m}-p_{f}\right)}^{2}}{4\;p\;q},$$ (2)

where *p*_m_ and *p*_f_ are the frequencies of *A* in mature males and females, *p* is its frequency in zygotes, and *q* = 1 − *p*.

Write the relative viabilities of genotypes *aa*, *Aa*, and *AA* in males as 1:: 1 + *h*_m_*S*_m_:: *S*_m_. The selection coefficient *S*_*m*_ and dominance coefficient *h*_*m*_ can be frequency-dependent, in which case these coefficients take their values at equilibrium (see below). Assuming that zygotes are at Hardy-Weinberg equilibrium, the allele frequency in adult males is

$$p_{m}\approx p+pq\;s_{m},$$ (3)

where *s*_*m*_ is the additive fitness effect of allele *A* in males, defined as

$$s_{m}=\left[p+\left(q-p\right)h_{m}\right]S_{m}.$$ (4)

The approximation of Eq (3) neglects terms of order $s_{m}^{2}$. An analogous expression gives the additive fitness effect of allele *A* in females, *s*_*f*_. The divergence between males and females is therefore

$$F_{ST}\approx \frac{1}{4}pq{\left(s_{m}-s_{f}\right)}^{2}.$$ (5)

We link selection to expression by assuming that when expression is low, the fitness effects can be approximated by the functions:

$$\begin{array}{c} s_{m}=a_{m}\;M\\ s_{f}=a_{\text{f}}\;F, \end{array}$$ (6)

where

$$\begin{array}{c} M=\text{ln}\left(m+1\right)\\ F=\text{ln}\left(f+1\right), \end{array}$$ (7)

and *m* and *f* are the absolute expression levels (e.g. number of transcripts per cell or in a tissue). The coefficients *a*_m_ and *a*_f_ reflect how sensitive selection is to the expression level. At a locus under sexually-antagonistic selection, these coefficients have opposite signs. The situation is sketched in Fig 3.

Eqs (6 and 7) have three desirable features. First, the fitness effect of an allele in a given sex is 0 if the gene is not expressed in that sex. Second, for values of *m* and *f* much larger than 1, changes in transcript abundance have proportional effects on the strength of selection. For example, a 10% increase in expression has the same effect on fitness regardless of whether there are 100 or 1,000 transcripts in the cell. Last, we will see below that the logarithmic scaling for expression used in Eq (7) leads to predictions from the model that can be directly compared to data.

We now assume that allele frequencies are at equilibrium under sexually-antagonistic viability selection, which implies that *s*_*m*_ = −*s*_*f*_. This assumption will be violated if fertility selection acts in addition to viability selection, but we do not anticipate that will systematically bias the predictions. (We note that an equilibrium is possible with any degree of dominance if selection is frequency-dependent.) The equilibrium condition and Eq (6) imply that

$$T=\left(\frac{a_{f}-a_{m}}{a_{f}+a_{m}}\right)D,$$ (8)

where *T* is the total expression and *D* is the difference in expression (sex-bias) between males and females:

$$\begin{array}{c} T=M+F\\ D=M-F. \end{array}$$ (9)

Substituting Eqs (6–9) into Eq (5) gives

$$F_{ST}\approx p\;q\;A\;D^{2},$$ (10)

where

$$A={\left(\frac{a_{m}a_{f}}{a_{m}+a_{f}}\right)}^{2}.$$ (11)

Eq (10) gives a pleasingly simple relationship between the divergence of allele frequencies between the males and females resulting from sexually-antagonistic selection, on the one hand, and the degree of sex-biased expression, on the other. Three quantities appear on the right side. First is the term *pq*, which is proportional to heterozygosity. The second is *A*, which is a measure of the potential for sexually-antagonistic selection. Last is *D*^2^, which is the square of the sex-bias in expression.

We want to express these results in terms of quantities that have been estimated. A natural measure of sex-biased is

$$\Delta =\frac{m-f}{m+f},$$ (12)

where *m* and *f* are the transcript abundances in males and females. The measure Δ is 0 when expression is unbiased, reaches a minimum value of Δ = −1 when a gene is expressed only in females, and a maximum value of Δ = 1 when it is expressed only in males. When bias is small and the absolute expression levels are much greater than 1, a Taylor expansion shows that

$$\Delta \approx \frac{1}{2}\text{ln}\left(\frac{m}{f}\right)\approx \frac{1}{2}D.$$ (13)

Substituting Eq (13) into Eq (10), we finally have

$$F_{\text{ST}}\approx 4\;p\;q\;A\;\Delta^{2},$$ (14)

which appears as Eq (1) in the main text.

This result has several implications. Divergence in allele frequencies between males and females (measured by *F*_ST_) is predicted be at a minimum when there is no sex bias in expression (Δ = 0). For small amounts of bias, either positive or negative, divergence should increase quadratically with |Δ|. We expect no divergence in male and female allele frequencies at the extremes of completely female-biased and completely male-biased expression (Δ = −1 and Δ = 1, respectively). That prediction follows because either male or female expression is then 0, and under our equilibrium assumption there will be no positive selection acting on the other sex that could generate a difference in allele frequencies in males and females.

To summarize, our model makes three predictions: *F*_ST_ will be 0 for a gene with unbiased expression (Δ = 0), it will increase quadratically with Δ for small amounts of male- and female-biased expression, and finally it will return to 0 for a gene that are expressed only in females or in males (Δ = −1 and Δ = 1). The results are shown graphically in Fig 4.

### The strength of sexually-biased selection

We estimated the strength of selection from the frequencies of alleles in females and males. Following the notations and assumptions described in previous sections, we assume that loci under sexually-antagonistic selection and are at equilibrium. That implies that the fitness effect of an allele in females is equal in magnitude and opposite in sign to its effect in males. The total numbers of alleles sampled from females and males are denoted *N*_f_ and *N*_m_, and the numbers of the minor allele observed are *n*_f_ and *n*_m_. We assume the samples are independent.

Denote the (unknown) frequency of the minor allele in zygotes at a given locus as *p*. Then likelihood of *s*_m_, the additive fitness effect in males as defined by Eq (4), is

$$L\left(s_{\text{m}},p\right)=B\left(n_{\text{f}};N_{\text{f}},p_{\text{f}}^{*}\right)B\left(n_{\text{m}};N_{\text{m}},p_{m}^{*}\right),$$ (15)

where *B*() is the binomial distribution, and the allele frequencies in the adults from which the alleles are sampled are

$$\begin{array}{c} p_{\text{m}}^{*}=p+p\;q\;s_{\text{m}}\\ p_{\text{f}}^{*}=p-p\;q\;s_{\text{m}}. \end{array}$$ (16)

(Eq (16) are approximations that assume *p q s*_m_ << 1.) The posterior probability of the allele’s fitness effect is

$$P_{\beta }\left(s_{\text{m}}\right)=\int_{0}^{1/2}L\left(s_{\text{m}},p\right)\text{Pr}\left(p\right)dp,$$ (17)

where Pr(*p*) is the prior probability density of *p*.

For Pr(*p*), we fit an *ad hoc* function to the distribution of minor allele frequencies observed in the YRI population. That function is:

$$\text{Pr}\left(p\right)=48.4\text{ Re}\left[\text{Exp}\left\{-35{\left(x-0.003\right)}^{0.7}\right\}\right]+3.59\left(0.5-x\right),$$ (18)

where Re[*x*] is the real part of *x*. The fit of this function to the data is shown in S1 Fig. In practice, we found that using a uniform prior distribution changed the estimates of *s*_m_ very little.

We evaluated posterior probability by numerically integrating Eq (17). We found the maximum *a posteriori* probability (MAP) estimate of *s*_m_ by numerically maximizing that function. The 95% credible interval was found by searching for the values of *s*_m_ that returned a posterior probability equal to 1/20 of the maximum probability.

S2 Fig shows the results for a sample of 15 genes. These were taken from loci with values of Δ that fall close to the maxima of the two Twin Peaks and whose values of *F*_ST_ averaged across all SNPs in the gene are typical for genes with that degree of sex-biased expression. For each of the genes in the sample, we chose the SNP with the largest *F*_ST_, reasoning that this SNP was more likely to be the actual target of sex-specific selection. All of the MAP estimates are very large $\left(\left|{\hat{s}}_{m}\right|\approx 1/2\right)$ but no estimate for *s*_m_ is significantly different from 0.

### The selection load

The selection load caused by sexually-antagonistic viability selection can be calculated by the following argument. The load is the average of the mortality incurred from sexually-antagonistic selection by males (μ_m_) and in females (μ_f_):

$$L_{SA}=\frac{1}{2}\left(\mu_{\text{m}}+\mu_{\text{f}}\right).$$ (19)

Following the notation used earlier, the relative viabilities of genotypes *aa*, *Aa*, and *AA* in males are 1 ∷ 1 + *h*_m_*S*_m_ ∷ *S*_m_, and analogous expressions give the relative viabilities in females. Assume Hardy-Weinberg equilibrium, and let *p* be the frequency of the allele that is beneficial to males. The mortalities are then:

$$\begin{array}{c} \mu_{\text{m}}\approx 2\;p\;q\;\left(1-h_{\text{m}}\right)S_{\text{m}}+q^{2}S_{\text{m}}\\ \mu_{\text{f}}\approx 2\;p\;q\;h_{\text{f}}S_{\text{f}}+p^{2}\;S_{\text{f}}. \end{array}$$ (20)

We now assume the population is at equilibrium. Then Eqs (3 and 4) imply that

$$\left[p+\left(q-p\right)h_{\text{m}}\right]S_{\text{m}}=\left[p+\left(q-p\right)h_{\text{f}}\right]S_{\text{f}},$$ (21)

which on rearranging gives

$$S_{\text{f}}=\left(\frac{p+\left(q-p\right)h_{\text{m}}}{p+\left(q-p\right)h_{\text{f}}}\right)S_{\text{m}}.$$ (22)

Substituting these results back into Eq (19) tells us that the sexually-antagonistic load is

$$L_{\text{SA}}=\left(\frac{p+q^{2}h_{\text{f}}-p^{2}h_{\text{m}}}{2\left[p+\left(q-p\right)h_{\text{f}}\right]}\right)S_{\text{m}}.$$ (23)

This result simplifies substantially when heterozygotes have intermediate fitness (*h*_m_ = *h*_f_ = 1/2):

$$L_{\text{SA}}=s,$$ (24)

where *s* is the additive fitness effect of allele *A*.

### Quantifying sex-specific selection using *F*_ST_

We downloaded SNP data on humans from the 1000 Genomes Project [16]. The sample includes more than 2000 individuals from 26 populations worldwide. The gene annotation file (GRCh38) was downloaded from Ensembl [43]. We filtered the data to include only SNPs in transcribed regions of autosomal protein coding genes. A SNP was excluded from a population if its minor allele appeared as a singleton (28% of all SNPs), and SNPs were removed from all populations if they were monomorphic in more than 5 populations (0.24% of all genes). The resulting dataset includes over 6 million SNPs in 17,839 autosomal genes. In a second series of analyses, we also included potential regulatory elements by analyzing all 34,060 transcripts profiled by the Genotype-Tissue Expression Project [17] and the 1kb upstream regions of protein-coding genes.

*F*_ST_ between the sexes and the genetic diversity in each population were estimated using the R package *PopGenome* [44]. The *F*_ST_ for each gene was summarized using the mean value for all SNPs inside that gene. The results were qualitatively unchanged when we used several alternative methods: the median or maximum *F*_ST_ (rather than the mean) of SNPs within a gene, including singleton SNPs, and alternative estimators for *F*_ST_. Likewise, results were not changed when we excluded autosomal genes with paralogs on the X or Y chromosome.

To test for significant *F*_ST_ between the sexes at individual SNPs, we applied the False Discovery Rate (FDR) approach using the qvalue package from R. Known SNPs inside genes with X or Y paralogs were removed. No SNP is significant at 5% FDR level. We further used Fisher’s method to combine *p*-values across multiple populations, and then calculated corresponding *q* value. Again, we were not able to find any significant SNP at the 5% FDR level. We conducted a power analysis to determine how likely that result is to occur under different strengths of selection (S1 File). We used parameter values corresponding to the Yoruban population (which is the most polymorphic and so most likely to show signals of sex-specific selection). We found that even if every one of the more than 3 million SNPs in this population experienced sexually-antagonistic selection with a selection coefficient as large as *s* = 0.25, there is about a 96% probability that none of them will show a significant *F*_ST_. It is therefore unsurprising that no SNP showed significant divergence.

To learn if sex-specific selection is more prevalent among genes with certain functions, we identified the SNP with the highest *F*_ST_ at each locus, since this SNP is more likely to be the target of selection. Gene set enrichment analysis was performed using online tools from the Database for Annotation, Visualization and Integrated Discovery [45]. Cell adhesion, synapses, neuron development, and several other annotation clusters are highly enriched among the loci that fall in the top 5% of *F*_ST_ values (DAVID scores greater than 7; S9 Table).

### Quantifying sex-biased gene expression

RNAseq data for gene expression in humans provided by the Genotype-Tissue Expression Project [17] was queried from the Gene Expression Atlas [46]. We used expression levels in ovaries measured in 6 females and levels in testes measured in 14 males. Relative sex bias in expression is measured as: Δ = (*m*–*f*) / (*m* + *f*), where *m* and *f* are the normalized transcript abundances in males and females. Our index of sex bias Δ is highly correlated (*r* = 0.95) with the familiar log_2_ ratio of male and female expression.

To determine if the results hold for tissues other than testes and ovaries, we added the data for all of the sex-specific somatic tissues available in the Genotype-Tissue Expression Project. For female-specific tissues, we averaged expression in the ovary, ectocervis, fallopian tubes, vagina, and uterus. For male-specific tissues, we averaged testes and prostate. The Twin Peaks pattern again appears, but with the left-hand peak shifted far to the left. This shift appears to be driven by the prostate: the original pattern reappears using the five female-specific tissues but only testes from males, but no pattern is seen using the five female-specific tissues and prostate. We believe that prostate does not provide a good proxy for gene expression in males: its expression profile is most similar to that of vagina, and is quite similar to other female-specific tissues [20]. We were unable to repeat our analyses with other somatic tissues because the Gene Expression Atlas only provides data on the average expression across both sexes.

### Reanalysis of fruit fly data

Gene expression data collected by Innocenti and Morrow [7] was downloaded from Gene Expression Omnibus (accession number GSE17013) and the probe annotation file from Ensemble. Following Innocenti and Morrow, the log_2_ ratio of male and female gene expression was estimated using the R *Bioconductor* packages [47]. Sex-biased expression was then transformed into our Δ statistic (described above). The list of candidate genes under sexually-antagonistic selection was taken from [7]. Results shown in the main text pertain to autosomal loci, but the Twin Peaks pattern also appears when sex-linked loci are included.

### Fitting curves

The relation between sex-specific selection and sex-biased expression was determined by first regressing *F*_ST_ onto Δ using polynomials. Each population was treated as a replicate. The optimal polynomial degree was determined using the Akaike information criterion [48] and likelihood ratio tests. Regressions were also fit with cubic splines using generalized additive models (GAM) implemented *mgcv* package [49] in R.

Following [50], we fit fourth degree polynomials to 10^5^ bootstrap samples of the original data. The Twin Peaks pattern were identified in a particular sample if it met three criteria: the fit was significant (*p* < 0.05 by ANOVA), the leading coefficient of the polynomial was negative, and the derivative of the polynomial had three real roots in the interval [−1, 1]. The last two criteria imply that there were two peaks (local maxima with a local minimum between them). A heat map was used to visualize the probability density for each value of *F*_ST_ as a function of Δ. The result is shown in Fig 1.

A permutation test was used to test the significance of the pattern we observed in the data. We permuted the values of Δ, fit a quartic polynomial by regression, and again used the three criteria to determine if the Twin Peaks pattern appeared. This procedure was repeated 10^5^ times.

The locations of the local minimum and the two local maxima for the polynomial regressions were found by using the *polyroot* function in R [51]. The 95% confidence intervals for these locations were estimated by bootstrapping the data 10^5^ times.

## Supporting Information

S1 Fig Fit of Eq (18) to the minor allele frequency distribution in the YRI population. (PDF)

S2 Fig Estimates of the sexually-antagonistic fitness effects at 15 loci. The loci were chosen at random from those with sex-biased expression levels corresponding to the Twin Peaks, and typical *F*_ST_ values for genes with that degree of expression bias. The maximum *a posteriori* probability (MAP) estimates are shown by the large horizontal line, and the 95% credible interval by the whiskers.
(PDF)

S3 Fig The relationship of *D*_a_ vs. Δ is shown by the best-fit cubic spline (blue line), and the 95% confidence interval is shown by the shaded area. (PDF)

S4 Fig The relationship of π vs Δ for is shown by the best-fit cubic spline (blue line), and the 95% confidence interval is shown by the shaded area. (PDF)

S5 Fig The probability of detecting significantly different allele frequencies in males and females at any SNP in the YRI population as a function of the strength of sexually-antagonistic selection. (PDF)

S1 Table A polynomial regression of *F*_ST_ on Δ is best fit by a 4^th^ degree polynomial. (PDF)

S2 Table A polynomial regression of *D*_a_ on Δ is best fit by a 4^th^ degree polynomial. (PDF)

S3 Table A polynomial regression of *F*_ST_ / (2*pq*) on Δ is best fit by a 4^th^ degree polynomial. (PDF)

S4 Table A polynomial regression of *F*_ST_ on Δ is best fit by a quadratic when Δ is small (–0.52 < Δ < 0.72). (PDF)

S5 Table A polynomial regression of *F*_ST_ on Δ is best fit by a 4^th^ degree polynomial when we including noncoding genes and regions 1kb upstream of coding regions. (PDF)

S6 Table A polynomial regression of *D*_a_ on Δ is best fit by a 4^th^ degree polynomial when we including noncoding genes and regions 1kb upstream of coding regions. (PDF)

S7 Table A polynomial regression of *F*_ST_ / (2*pq*) on Δ is best fit by a 4^th^ degree polynomial when we including noncoding genes and regions 1kb upstream of coding regions. (PDF)

S8 Table A polynomial regression of *F*_ST_ on Δ is best fit by a quadratic when Δ is small (–0.52 < Δ < 0.72) when we including noncoding genes and regions 1kb upstream of coding regions. (PDF)

S1 File The details of a power analysis that calculates the probability of observing a statistically significant difference between allele frequencies in males and females that results from sexually-antagonistic selection. (PDF)

## References

[1] Darwin C. The descent of man, and selection in relation to sex. London: Murray; 1871.

[2] Lande R. Sexual dimorphism, sexual selection, and adaptation in polygenic characters. Evolution. 1980;34: 292–305. 10.2307/2407393 28563426

[3] Rice WR. Sex chromosomes and the evolution of sexual dimorphism. Evolution. 1984;38: 735–742. 10.2307/2408385 28555827

[4] Arnqvist G, Rowe L. Sexual conflict. Princeton University Press; 2005.

[5] Vicoso B, Charlesworth B. Evolution on the X chromosome: unusual patterns and processes. Nat Rev Genet. 2006;7: 645–653. 10.1038/nrg1914 16847464

[6] Ellegren H, Parsch J. The evolution of sex-biased genes and sex-biased gene expression. Nat Rev Genet. 2007;8: 689–698. 10.1038/nrg2167 17680007

[7] Innocenti P, Morrow EH. The sexually antagonistic genes of Drosophila melanogaster. PLoS Biol. 2010;8: e1000335 10.1371/journal.pbio.1000335 20305719 PMC2838750

[8] van Doorn GS. Intralocus sexual conflict. Ann N Y Acad Sci. 2009;1168: 52–71. 10.1111/j.1749-6632.2009.04573.x 19566703

[9] Bonduriansky R, Chenoweth SF. Intralocus sexual conflict. Trends Ecol Evol. 2009;24: 280–288. 10.1016/j.tree.2008.12.005 19307043

[10] Rice WR. Nothing in genetics makes sense except in light of genomic conflict. Annu Rev Ecol Evol Syst. 2013;44: 217–237. 10.1146/annurev-ecolsys-110411-160242

[11] Charlesworth D, Charlesworth B. Sex differences in fitness and selection for centric fusions between sex-chromosomes and autosomes. Genet Res. 1980;35: 205–214. 10.1017/S0016672300014051 6930353

[12] van Doorn GS, Kirkpatrick M. Turnover of sex chromosomes induced by sexual conflict. Nature. 2007;449: 909–912. 10.1038/nature06178 17943130

[13] van Doorn GS, Kirkpatrick M. Transitions between male and female heterogamety caused by sex-antagonistic selection. Genetics. 2010;186: 629–645. 10.1534/genetics.110.118596 20628036 PMC2954476

[14] Connallon T, Cox RM, Calsbeek R. Fitness consequences of sex-specific selection. Evolution. 2010;64: 1671–1682. 10.1111/j.1558-5646.2009.00934.x 20050912

[15] Kirkpatrick M, Guerrero RF. Signatures of sex-antagonistic selection on recombining sex chromosomes. Genetics. 2014;197: 531–541. 10.1534/genetics.113.156026 24578352 PMC4063913

[16] The 1000 Genomes Project Consortium. A global reference for human genetic variation. Nature. 2015;526: 68–74. 26432245 10.1038/nature15393 PMC4750478

[17] Lonsdale J, Thomas J, Salvatore M, Phillips R, Lo E, Shad S, et al The Genotype-Tissue Expression (GTEx) project. Nat Genet. 2013;45: 580–585. 10.1038/ng.2653 23715323 PMC4010069

[18] Wright AE, Mank JE. The scope and strength of sex-specific selection in genome evolution. J Evol Biol. 2013;26: 1841–1853. 10.1111/jeb.12201 23848139 PMC4352339

[19] Harrison PW, Wright AE, Zimmer F, Dean R, Montgomery SH, Pointer MA, et al Sexual selection drives evolution and rapid turnover of male gene expression. Proc Natl Acad Sci. 2015;112: 4393–4398. 10.1073/pnas.1501339112 25831521 PMC4394296

[20] Melé M, Ferreira PG, Reverter F, DeLuca DS, Monlong J, Sammeth M, et al The human transcriptome across tissues and individuals. Science. 2015;348: 660–665. 10.1126/science.aaa0355 25954002 PMC4547472

[21] Nei M, Li WH. Mathematical model for studying genetic variation in terms of restriction endonucleases. Proc Natl Acad Sci. 1979;76: 5269–5273. 291943 10.1073/pnas.76.10.5269 PMC413122

[22] Cruickshank TE, Hahn MW. Reanalysis suggests that genomic islands of speciation are due to reduced diversity, not reduced gene flow. Mol Ecol. 2014;23: 3133–3157. 10.1111/mec.12796 24845075

[23] Kong A, Thorleifsson G, Stefansson H, Masson G, Helgason A, Gudbjartsson DF, et al Sequence variants in the RNF212 gene associate with genome-wide recombination rate. Science. 2008;319: 1398–1401. 10.1126/science.1152422 18239089

[24] Sandor C, Li W, Coppieters W, Druet T, Charlier C, Georges M. Genetic variants in REC8, RNF212, and PRDM9 influence male recombination in cattle. PLoS Genet. 2012;8: e1002854 10.1371/journal.pgen.1002854 22844258 PMC3406008

[25] Heid IM, Jackson AU, Randall JC, Winkler TW, Qi L, Steinthorsdottir V, et al Meta-analysis identifies 13 new loci associated with waist-hip ratio and reveals sexual dimorphism in the genetic basis of fat distribution. Nat Genet. 2010;42: 949–960. 10.1038/ng.685 20935629 PMC3000924

[26] Singh D. Adaptive significance of female physical attractiveness: role of waist-to-hip ratio. J Pers Soc Psychol. 1993;65: 293 8366421 10.1037//0022-3514.65.2.293

[27] Gilks WP, Abbott JK, Morrow EH. Sex differences in disease genetics: evidence, evolution, and detection. Trends Genet. 2014;30: 453–463. 10.1016/j.tig.2014.08.006 25239223

[28] DeGiorgio M, Lohmueller KE, Nielsen R. A model-based approach for identifying signatures of ancient balancing selection in genetic data. PLoS Genet. 2014;10: e1004561 10.1371/journal.pgen.1004561 25144706 PMC4140648

[29] Connallon T, Clark AG. Balancing selection in species with separate sexes: insights from Fisher’s geometric model. Genetics. 2014; genetics.114.165605. 10.1534/genetics.114.165605 PMC4096376 24812306

[30] Keightley PD. Rates and fitness consequences of new mutations in humans. Genetics. 2012;190: 295–304. 10.1534/genetics.111.134668 22345605 PMC3276617

[31] Lucotte EA, Laurent R, Heyer E, Ségurel L, Toupance B. Detection of allelic frequency differences between the sexes in humans: a signature of sexually antagonistic selection. Genome Biol Evol. 2016; evw090. 10.1093/gbe/evw090 PMC4898804 27189992

[32] Consortium TIH 3. Integrating common and rare genetic variation in diverse human populations. Nature. 2010;467: 52–58. 10.1038/nature09298 20811451 PMC3173859

[33] Chippindale AK, Gibson JR, Rice WR. Negative genetic correlation for adult fitness between sexes reveals ontogenetic conflict in Drosophila. Proc Natl Acad Sci. 2001;98: 1671–1675. 10.1073/pnas.98.4.1671 11172009 PMC29315

[34] Mank JE, Hultin-Rosenberg L, Zwahlen M, Ellegren H. Pleiotropic constraint hampers the resolution of sexual antagonism in vertebrate gene expression. Am Nat. 2008;171: 35–43. 10.1086/523954 18171149

[35] Poissant J, Wilson AJ, Coltman DW. Sex-specific genetic variance and the evolution of sexual dimorphism: a systematic review of cross-sex genetic correlations. Evolution. 2010;64: 97–107. 10.1111/j.1558-5646.2009.00793.x 19659596

[36] Griffin RM, Dean R, Grace JL, Rydén P, Friberg U. The shared genome is a pervasive constraint on the evolution of sex-biased gene expression. Mol Biol Evol. 2013;30: 2168–2176. 10.1093/molbev/mst121 23813981

[37] Delcourt M, Blows MW, Rundle HD. Sexually antagonistic genetic variance for fitness in an ancestral and a novel environment. Proc R Soc Lond B Biol Sci. 2009;276: 2009–2014. 10.1098/rspb.2008.1459 PMC2677236 19324806

[38] Rice WR, Chippindale AK. Intersexual ontogenetic conflict. J Evol Biol. 2001;14: 685–693. 10.1046/j.1420-9101.2001.00319.x

[39] Rice WR, Holland B. The enemies within: intergenomic conflict, interlocus contest evolution (ICE), and the intraspecific Red Queen. Behav Ecol Sociobiol. 1997;41: 1–10. 10.1007/s002650050357

[40] Ranz JM, Castillo-Davis CI, Meiklejohn CD, Hartl DL. Sex-dependent gene expression and evolution of the drosophila transcriptome. Science. 2003;300: 1742–1745. 10.1126/science.1085881 12805547

[41] Zhang Y, Sturgill D, Parisi M, Kumar S, Oliver B. Constraint and turnover in sex-biased gene expression in the genus Drosophila. Nature. 2007;450: 233–237. 10.1038/nature06323 17994089 PMC2386141

[42] Jiang Z-F, Machado CA. Evolution of sex-dependent gene expression in three recently diverged species of Drosophila. Genetics. 2009;183: 1175–1185. 10.1534/genetics.109.105775 19720861 PMC2778969

[43] Cunningham F, Amode MR, Barrell D, Beal K, Billis K, Brent S, et al Ensembl 2015. Nucleic Acids Res. 2015;43: D662–D669. 10.1093/nar/gku1010 25352552 PMC4383879

[44] Pfeifer B, Wittelsbürger U, Onsins SER, Lercher MJ. PopGenome: An efficient swiss army knife for population genomic analyses in R. Mol Biol Evol. 2014; msu136. 10.1093/molbev/msu136 PMC4069620 24739305

[45] Huang DW, Sherman BT, Lempicki RA. Systematic and integrative analysis of large gene lists using DAVID bioinformatics resources. Nat Protoc. 2008;4: 44–57. 10.1038/nprot.2008.211 19131956

[46] Kapushesky M, Emam I, Holloway E, Kurnosov P, Zorin A, Malone J, et al Gene Expression Atlas at the European Bioinformatics Institute. Nucleic Acids Res. 2010;38: D690–D698. 10.1093/nar/gkp936 19906730 PMC2808905

[47] Gentleman RC, Carey VJ, Bates DM, Bolstad B, Dettling M, Dudoit S, et al Bioconductor: open software development for computational biology and bioinformatics. Genome Biol. 2004;5: R80 10.1186/gb-2004-5-10-r80 15461798 PMC545600

[48] Burnham KP, Anderson DR. Model selection and multimodel inference: a practical information-theoretic approach. Springer Science & Business Media; 2003.

[49] Wood SN. mgcv: GAMs and generalized ridge regression for R. R News. 2001;1: 20–25.

[50] Hsiang SM. Visually-weighted regression [Internet]. Rochester, NY: Social Science Research Network; 2013 5 Report No.: ID 2265501. Available: http://papers.ssrn.com/abstract=2265501

[51] Team RC. R: A language and environment for statistical computing. Vienna, Austria; 2014 URL http://www.R-Proj.Org. 2015;

