# Supplementary figures and images for: Sex-Specific Selection and Sex-Biased Gene Expression in Humans and Flies
Source: PLoS Genet. 2016 Sep 22;12(9):e1006170. doi: 10.1371/journal.pgen.1006170 (PMC5033347; doi:10.1371/journal.pgen.1006170)

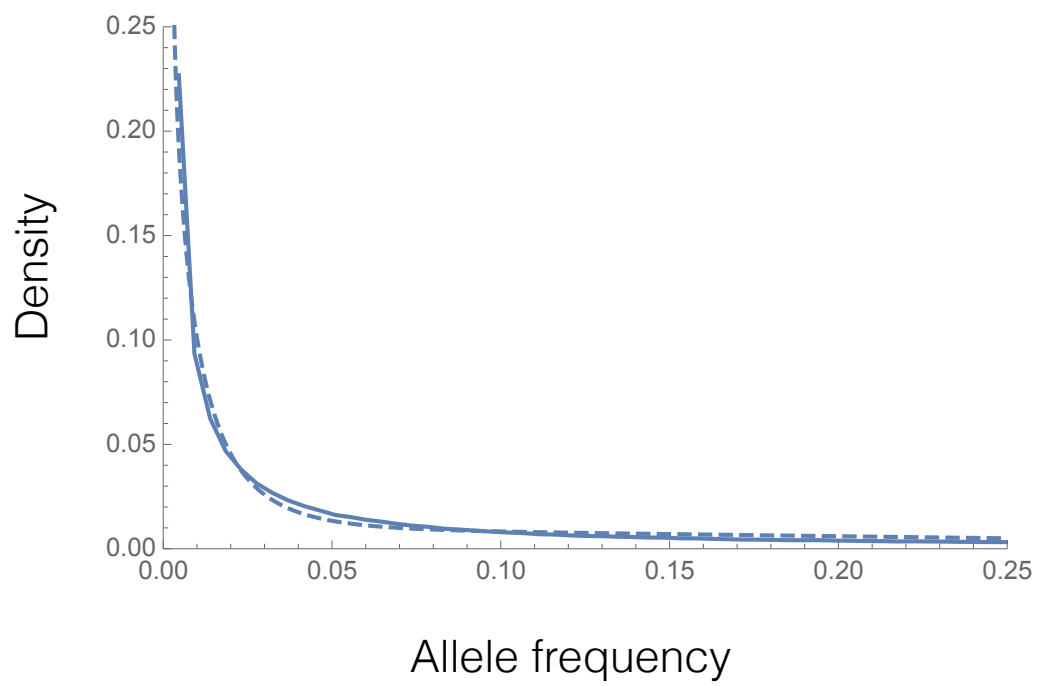

Supplement: S1 Fig — (PDF) [file pgen.1006170.s001.pdf]

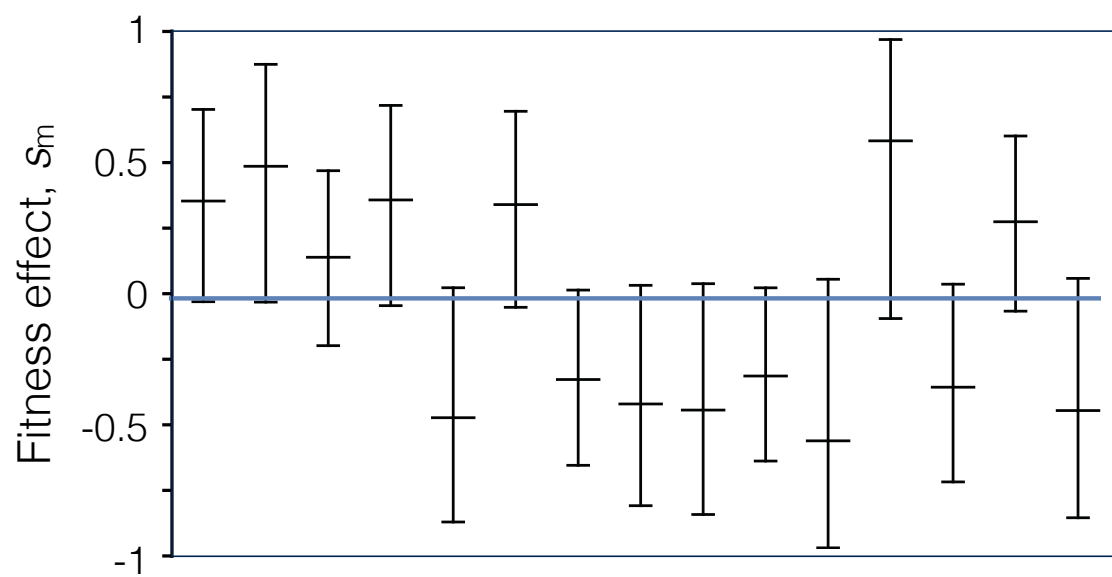

Supplement: S2 Fig — The loci were chosen at random from those with sex-biased expression levels corresponding to the Twin Peaks, and typical FST values for genes with that degree of expression bias. The maximum a posteriori probability (MAP) estimates are shown by the large horizontal line, and the 95% credible interval by the whiskers. (PDF) [file pgen.1006170.s002.pdf]

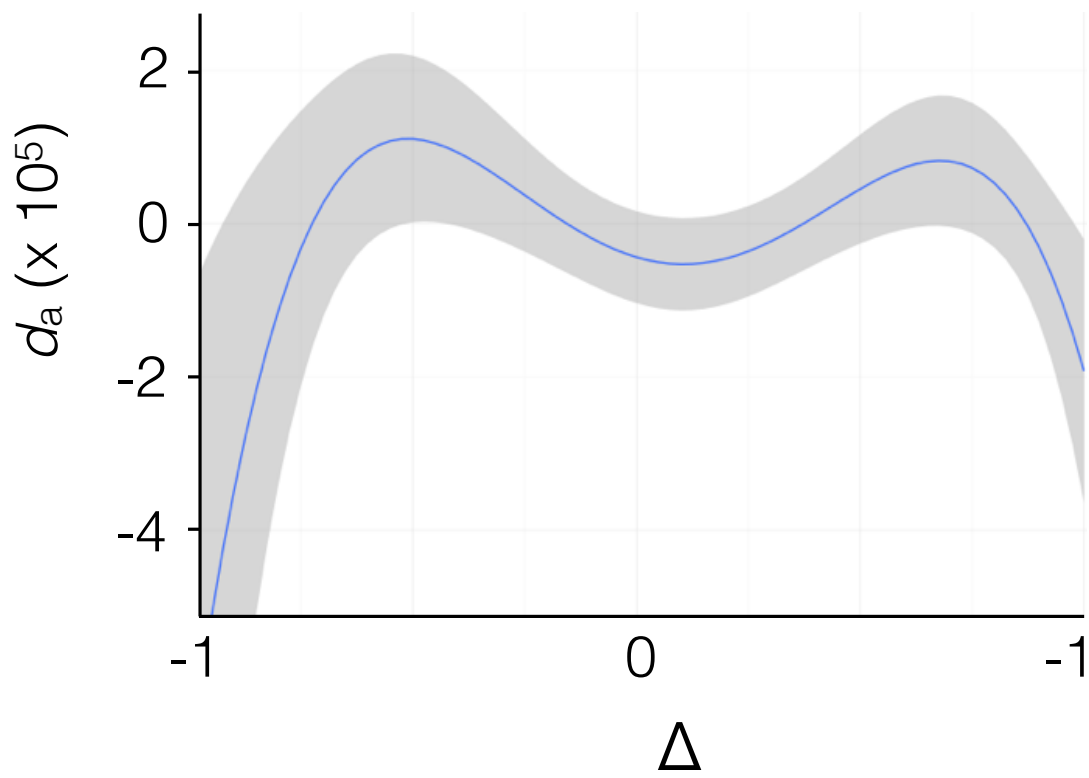

Supplement: S3 Fig — (PDF) [file pgen.1006170.s003.pdf]

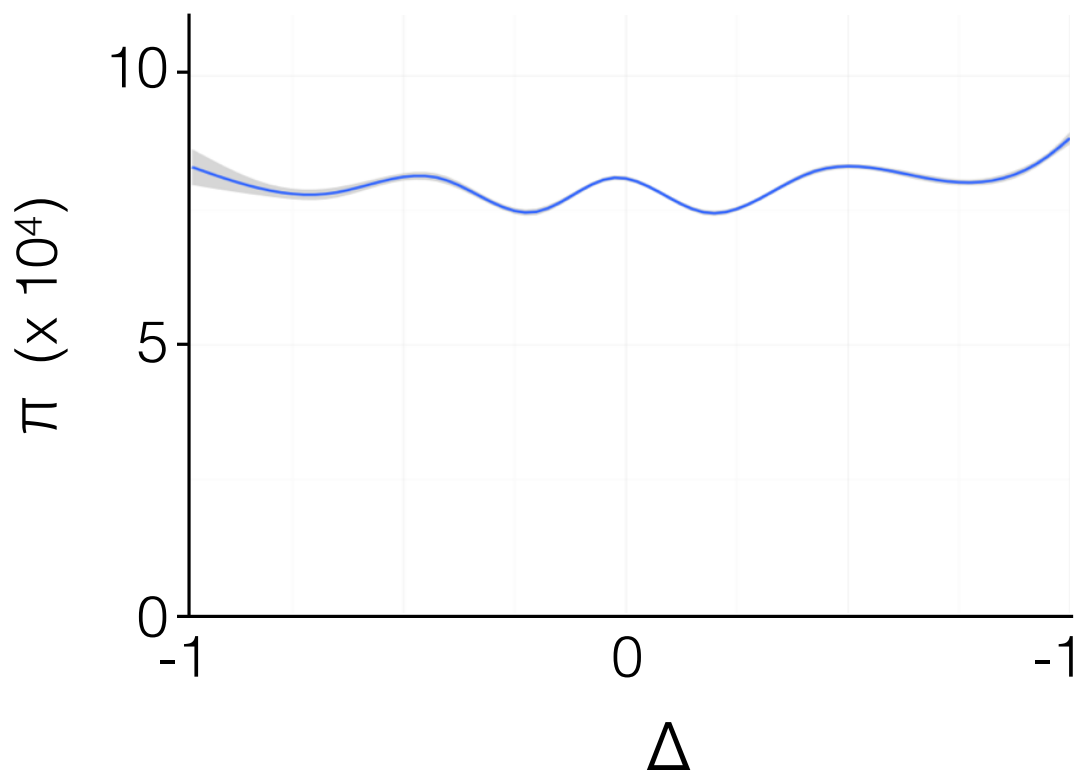

Supplement: S4 Fig — (PDF) [file pgen.1006170.s004.pdf]

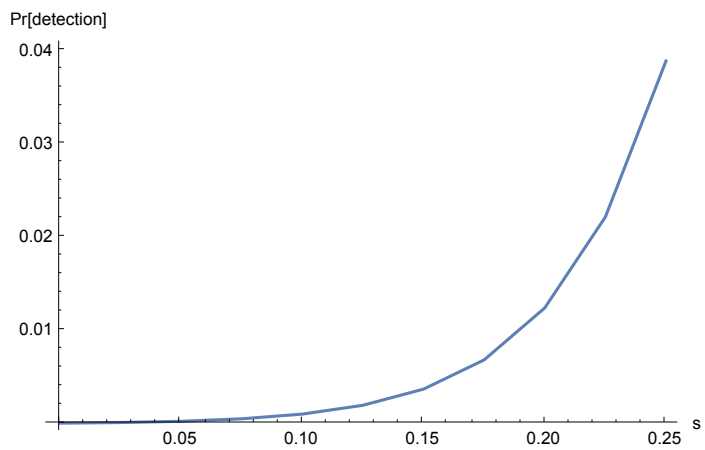

Supplement: S5 Fig — (PDF) [file pgen.1006170.s005.pdf]
