# Supplementary material for: Sex-Specific Selection and Sex-Biased Gene Expression in Humans and Flies
Source: PLoS Genet. 2016 Sep 22;12(9):e1006170. doi: 10.1371/journal.pgen.1006170 (PMC5033347; doi:10.1371/journal.pgen.1006170)
Supplement: S1 Table — (PDF) [file pgen.1006170.s006.pdf]

Degree	AIC	Likelihood ratio test p-value (vs. 4th degree model)
0	4	0.019
1	6	0.008
2	8	0.003
3	10	0.0004
4	-	-
5	2	0.64
6	4	0.88
