# Supplementary material for: Sex-Specific Selection and Sex-Biased Gene Expression in Humans and Flies
Source: PLoS Genet. 2016 Sep 22;12(9):e1006170. doi: 10.1371/journal.pgen.1006170 (PMC5033347; doi:10.1371/journal.pgen.1006170)
Supplement: S7 Table — (PDF) [file pgen.1006170.s012.pdf]

Models	AIC	Likelihood ratio test p-value (vs. 4th degree model)
0	8	0.003
1	10	0.001
2	9	0.002
3	10	0.001
4	-	-
5	2	0.54
6	2	0.36
