# Supplementary material for: Sex-Specific Selection and Sex-Biased Gene Expression in Humans and Flies
Source: PLoS Genet. 2016 Sep 22;12(9):e1006170. doi: 10.1371/journal.pgen.1006170 (PMC5033347; doi:10.1371/journal.pgen.1006170)
Supplement: S8 Table — (PDF) [file pgen.1006170.s013.pdf]

Models	AIC	Likelihood ratio test p-value (vs. 4th degree model)
0	23	<0.001
1	25	<0.001
2	-	-
3	1	0.37
4	3	0.52
