# Supplementary material for: Sex-Specific Selection and Sex-Biased Gene Expression in Humans and Flies
Source: PLoS Genet. 2016 Sep 22;12(9):e1006170. doi: 10.1371/journal.pgen.1006170 (PMC5033347; doi:10.1371/journal.pgen.1006170)
Supplement: S1 File — (PDF) [file pgen.1006170.s014.pdf]

S1 File: Power analysis

We would like to determine the probability of detecting a significant difference in allele frequencies between males and females at one or more of a set of loci (or SNPs). Here we assume that the allele frequencies diverge under sexually-antagonistic selection, in which selection favoring one allele is balanced by equally strong selection against that allele in the other sex. For simplicity, we assume that the loci have equal allele frequencies and intensities of selection. Our criterion for significance is based on a Bonferroni correction for multiple statistical tests.

The probability of detecting a significant allele frequency difference at one or more loci of n loci is

$$P_n = 1 - [1 - P_1]^n \quad \text{(S1)}$$

where P_1 is the probability of detecting a significant difference at a single locus after the Bonferroni correction. This last probability is

$$P_1 = \sum_{m=0}^{N_m} \sum_{f=0}^{N_f} B(m;\ N_m,\ p_m)\, B(f;\ N_f,\ p_f)\, S(m,\ N_m,\ f,\ N_f). \quad \text{(S2)}$$

Here $B(i;\ N,\ p)$ is the binomial probability of i successes in N trials each with probability p, N_m and N_f are the number of alleles sampled from males and females, and p_m and p_f are the minor allele frequencies in males and females after viability selection acts. $S(n_1,\ N_1,\ n_2,\ N_2)$ is an indicator variable that take a value of 1 if our test statistic is significant for a contingency table with entries $(n_1,\ N_1,\ n_2,\ N_2)$, and is 0 otherwise. For a nominal significance level of α, the Bonferroni correction for n loci has us evaluate $S()$ using a significance value of α/n. For ease of computation, we used χ^2 as our test statistic.

For simplicity, we assume genic (multiplicative) selection in which the fitnesses of genotypes $A_0 A_0$, $A_0 A_1$, and $A_1 A_1$ are $1 :: 1 + s :: (1 + s)^2$ in males. The allele frequencies in mature males and females are

$$p_m = p + \Delta p, \quad p_f = p - \Delta p,$$

where

$$\Delta p = \frac{s\,p\,(1 - p)}{1 + s\,p}. \quad \text{(S3)}$$

We implimented these calculations in *Mathematica* (Wolfram Research 2015).

We evaluated the results with parameters corresponding to our sample from the YRI population (which is the most polymorphic): a minor allele frequency of $p = 0.091$, $n = 3{,}171{,}261$ loci, $N_f = 112$ alleles sample from females, and $N_m = 104$ alleles sampled from males. The probabilities of detecting a significant difference in allele frequencies between the sexes at one or more loci (P_n) are shown for selection coefficients ranging from $s = 0$ to 0.25 in S5 Figure. We see that even if sexually-antagonistic selection is implausibly strong and common, with a selection coefficient of $s = 0.25$ acting on every one of more than 3 million SNPs, there is less than a 4% chance of detecting a significant difference.
